# Factors associated with antenatal depression among women attending antenatal care at Mubende Regional Referral Hospital: a cross-sectional study

**DOI:** 10.1186/s12905-024-03031-0

**Published:** 2024-03-25

**Authors:** Musa Kasujja, Samuel Omara, Nasifu Senkungu, Shamim Ndibuuza, Joseph Kirabira, Usman Ibe, Lyse Barankunda

**Affiliations:** 1 Kampala International University Western Campus, Kampala, Uganda; 2 Nile International Hospital, Jinja, Uganda; 3 Arahmah Medical Centre, Masaka, Uganda

**Keywords:** Depression during pregnancy, Antenatal, Prenatal, Depression

## Abstract

**Introduction:**

This study aimed to investigate the prevalence, severity, and factors associated with antenatal depression among women receiving antenatal care at Mubende Regional Referral Hospital (MRRH) in Uganda. Antenatal depression is a critical concern for maternal and child well-being, as it is associated with adverse outcomes such as preterm birth, abortion, low birth weight, and impaired maternal-infant bonding. Despite several international guidelines recommending routine screening for antenatal depression, local Ugandan guidelines often overlook this essential aspect of maternal care.

**Methods:**

A cross-sectional study involving 353 pregnant women utilized the Patient Health Questionnaire 9 (PHQ-9) to assess antenatal depression. Participants were categorized as having antenatal depression if their total PHQ-9 score was ≥ 5 and met the Diagnostic and Statistical Manual of Mental Disorders, Fourth Edition (DSM-IV) criteria for either major or minor depression. Psychosocial demographic and obstetric characteristics were recorded. Logistic regression analysis identified factors linked to antenatal depression.

**Results:**

The burden of antenatal depression was notably high, affecting 37.68% of the participants. Among those with antenatal depression, the majority exhibited mild symptoms 94 (70.68%). The significant factors associated with antenatal depression, revealed by multivariate analysis, included younger age (≤ 20 years), older age (≥ 35 years), history of domestic violence, alcohol use, gestational age, history of abortion, history of preeclampsia, and unplanned pregnancies.

**Conclusion:**

This study revealed a significantly high prevalence of antenatal depression, emphasizing its public health importance. Most cases were classified as mild, emphasizing the importance of timely interventions to prevent escalation. The identified risk factors included age, history of domestic violence, alcohol use, first-trimester pregnancy, abortion history, previous preeclampsia, and unplanned pregnancy.

## Introduction

### Background

Antenatal depression is characterized by a nonpsychotic depressed mood disorder lasting two weeks or more, marked by feelings of low mood, loss of interest in previously enjoyable activities, low self-esteem, guilt, low energy, and suicidal thoughts, occurring during pregnancy [1]. Various stressors during pregnancy, such as unintended pregnancy [2, 3], pregnancy complications [4], chronic disease [5], previous mental illness [6], fear of childbirth [7], gestational age [8], and maternal age [9], increase the risk of developing depression.

Depression during pregnancy elevates maternal noradrenaline and cortisol levels, reducing uterine blood flow and leading to adverse obstetric and neonatal outcomes. These outcomes include spontaneous abortion, hypertensive disorders, antepartum hemorrhage, intrauterine growth restriction, prematurity, low birth weight, low Apgar scores, neonatal intensive care unit admissions, and postnatal depression [10–12]. Antenatal depression is also associated with risky behaviors such as substance abuse (e.g., alcoholism and smoking), reduced healthcare service uptake, poor appetite, and suicide, further compromising fetal and maternal well-being [6].

Despite being the most prevalent mental health problem during pregnancy, antenatal depression often goes undetected and untreated, potentially leading to adverse obstetric and neonatal outcomes [11, 13, 14]. In Uganda, previous studies have reported a high prevalence of antenatal depression (35.8%) [15]. However, maternal and child health programs in the country primarily focus on improving the nutritional and physical well-being of mothers, neglecting their mental health needs.

Current perinatal care guidelines established by the American College of Obstetricians and Gynecologists advocate routine screening for depression for all pregnancies [16]. Other international standards, such as the World Health Organization (WHO) [17], National Institute for Health and Care Excellence (NICE) [18], and (RCOG) [19], also advocate for the use of consistent, validated tests to screen for antenatal depression during antenatal care. However, local clinical guidelines in Uganda lack a standard protocol for screening for antenatal depression [20]. This absence of screening as part of standard antenatal care may contribute to increased prevalence, inadequate detection and treatment, and serious consequences for both mothers and babies.

Given the importance of maternal mental health, this study aimed to provide evidence on the prevalence, severity, and factors associated with antenatal depression among women attending antenatal care at Mubende Regional Referral Hospital (MRRH).

### Specific objectives:

- To determine the prevalence of antenatal depression among women attending antenatal care at Mubende Regional Referral Hospital.
- To determine the factors associated with antenatal depression among women attending antenatal care at Mubende Regional Referral Hospital.

## Materials and methods

### Study design

Cross-sectional study.

### Study setting

This study was conducted during August 2023 in the antenatal care section of MRRH, a publicly funded tertiary healthcare center situated in the Mubende town council, Kyaterekera Parish, Mubende district in Uganda's central region.

### Eligibility criteria

#### Inclusion criteria

All pregnant women who were attending antenatal care at MRRH during the study period.

#### Exclusion criteria

Women who experienced obstetric emergencies or medical illnesses such as eclampsia, ruptured ectopic, or epilepsy, for whom urgent attention was needed. Women who had a severe mental health problem associated with lack of insight and therefore couldn't respond to the interviewer-administered questionnaire. For example, schizophrenia and bipolar affective disorder. Women who were receiving psychiatric treatment and/or antidepressant medications.

### Study procedure

At the beginning of each antenatal care clinic session, the researcher delivered an educational talk outlining the study's objectives, inclusion and exclusion criteria, and potential benefits. A consecutive sampling procedure was then employed. Research assistants obtained a list of eligible women attending the clinic each day and approached each woman to participate in the study. Women who agreed to participate were provided with appropriate privacy and confidentiality. The researcher and two research assistants administered a structured questionnaire to each participant individually, away from the hearing of others. Informed consent was obtained from all participants in the language they understood before administering the questionnaire. The participants were required to respond to each question they were asked about individually.

### Study variables and instruments

Data on psychosocial demographic factors and obstetric factors were collected using interviewer-administered questionnaires, while data on the prevalence and severity of antenatal depression were collected using the Patient Health Questionnaire-9 (PHQ-9).

The PHQ-9 is a self-reported tool consisting of nine items widely used for screening depressive symptoms experienced over the past two weeks [21]. It has been validated for perinatal research in Africa, specifically in Ethiopia [22], and in primary healthcare settings in Uganda [23]. The nine items of the PHQ-9 directly align with the diagnostic criteria for major depressive disorder outlined in the Diagnostic and Statistical Manual of Mental Disorders, Fourth Edition (DSM-IV) [24].

Each item represents a set of symptoms and is scored on a Likert scale based on the frequency of symptoms experienced over the past two weeks, ranging from 0 (not at all), 1 (several days), 2 (more than half the days), and 3 (nearly every day). The total PHQ-9 score ranges from 0 to 27, indicating the severity of depressive symptoms.

In this study, women who scored 5 or higher on the PHQ-9 and met the DSM-IV criteria for either major or minor depression on the questionnaire were categorized as having antenatal depression.

The DSM-IV criteria for major depression require the presence of five or more depressive symptoms experienced for more than half the days for at least 2 weeks, with at least one of these symptoms being a depressed mood or loss of interest in previously enjoyable activities. Conversely, the criteria for minor depression require the presence of at least two but fewer than five depressive symptoms experienced for more than half the days for at least 2 weeks, with at least one of these symptoms being a depressed mood or loss of interest in previously enjoyable activities [25].

Consistent with research conducted elsewhere in Africa [26, 27] and in Uganda [23, 28], we categorized depression into mild (PHQ score 5–9), moderate (PHQ score 10–14), or severe (PHQ score > 15) depression. It is important to note that the term "depression" in this context does not denote a clinical diagnosis but rather the outcome of a screening procedure in an epidemiological study with the properties mentioned earlier. In low-resource settings in Africa, the PHQ-9 has demonstrated superiority over other common depression screening measures, exhibiting better test–retest reliability than the Edinburgh Postnatal Depression Scale (EPDS) and better criterion validity than either the EPDS or the Self-Reporting Questionnaire-20 (SRQ-20) [29].

### Data quality control

To guarantee that the questionnaires were appropriate, simple English was used, and the use of technical terms was limited. The questionnaires were translated into Luganda and back-translated for accuracy. Translated questionnaires were read to English-naive participants. The researcher monitored the data collection process, and immediate cross-checking of completed questionnaires was conducted to ensure accuracy and completeness.

### Sample size calculation

The sample size was estimated using the statistical equation proposed by Kish Leslie (1965): $n=\frac{{Z}^{2}PQ}{{D}^{2}}$

Where $n$ = desired sample size, $Z$= the standard normal deviation at the 95% confidence level, $Z$=1.96, and $P$ = proportion of the target population estimated to have the specific characteristic (female attending antenatal care who has antenatal depression). which was found to be 35.8% in Uganda [15]. $Q$= (1- $P$) = proportion of the population without the characteristic. $D$ = precision of the study (the degree of accuracy desired). In this case, $D$ = 5% was used.

By substituting the given values into the equation, the sample size $(n)$ was calculated to be 353.

### Data analysis

Objective one involved summarizing the prevalence of antenatal depression as frequency and percentage with 95% confidence intervals. The severity of antenatal depression was categorized based on PHQ-9 scores. Objective two assessed factors associated with antenatal depression using binary logistic regression. Statistical significance was set at a *p*-value less than 0.05. Results were presented through pie charts, tables, bar graphs, and discussion for effective interpretation.

## Results

### Study conduct and participant selection

**Fig. 1:**
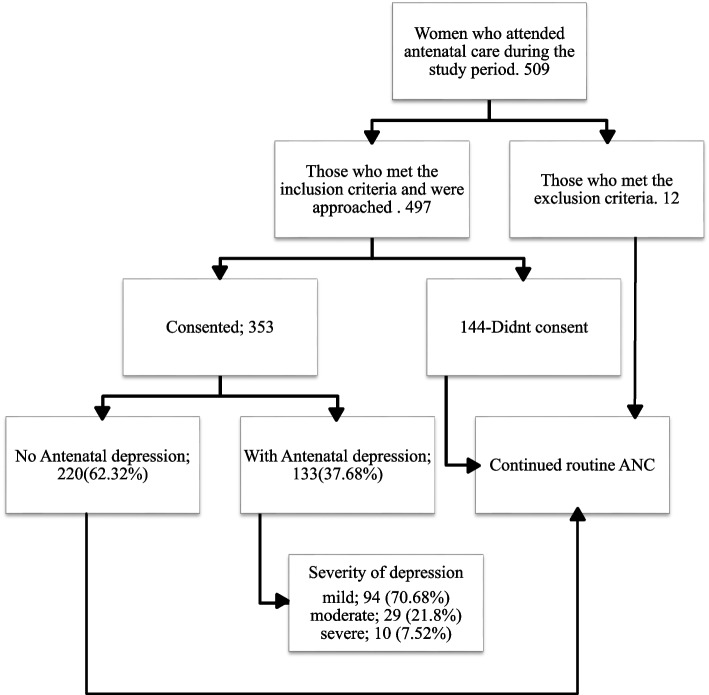
Study flowchart

This study was conducted in the antenatal care unit of the MRRH during the timeframe encompassing August 2023. A total of 509 women were given an educational talk by the researcher regarding the study’s objectives, inclusion and exclusion criteria, and benefits. Women who met the exclusion criteria or did not provide consent were excluded from participation. The study flow is shown in Fig. 1.

### Descriptive statistics for participant characteristics

Table 1 presents the psychosocial demographic characteristics of the participants.

**Table 1:** Psychosocial demographic characteristics, *N* = 353

Psycho-social demographic characteristics		Frequency (*n*)	Percentage (%)
**Age**	17 to 20	94	26.63
	21 to 34	220	62.32
	35 and above	39	11.05
**Marital status**	Married/Cohabiting	328	92.92
	Single/Separated	25	7.08
**Education**	Primary	218	61.76
	Secondary & Tertiary	135	38.24
**Employment**	Employed	190	53.82
	Unemployed	163	46.18
**Household Income (UGX)**	Less than 500,000	195	55.24
	More than 500,000	158	44.76
**Family history of mental illness**	No	303	85.84
	Yes	50	14.16
**History of domestic violence**	No	329	93.2
	Yes	24	6.8
**Alcohol use**	No	322	91.22
	Yes	31	8.78

Table 2 presents the obstetric characteristics of the research population.

**Table 2:** Obstetric characteristics of the participants (*N* = 353)

Obstetric characteristics		Frequency (n)	Percentage (%)
**Gestational Age**	Less than 13 weeks	46	13.03
	13 to 28 weeks	130	36.83
	More than 28 weeks	177	50.14
**Previous history of Preeclampsia**	No	330	93.48
	Yes	23	6.52
**History of abortion**	No	299	84.7
	Yes	54	15.3
**History of a Stillbirth**	No	335	94.9
	Yes	18	5.1
**Pregnancy Planning**	No (Unplanned)	196	55.52
	Yes (Planned)	157	44.48

### Prevalence of antenatal depression

Table 3 illustrates that of the 353 participants, 133 (37.68%) were classified as having depression, while 220 (62.32%) were identified as not having depression.

**Table 3:** Prevalence of antenatal depression

Category	Frequency(n)	Percentage (%)	95% CI
Depression	133	37.68	(32.60 – 42.96)
No depression	220	62.32	(57.04—67.40)
Total	353	100.00	

### Severity of antenatal depression

**Fig. 2:**
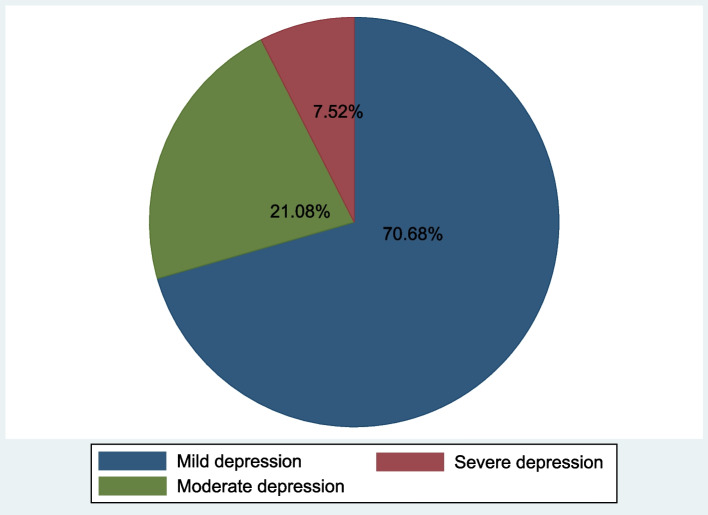
Pie chart showing the distribution of antenatal depression severity

Figure 2 illustrates the distribution of antenatal depression severity among women with antenatal depression. Out of the 133 participants with antenatal depression, 94 (70.68%) had mild depression, 29 (21.80%) had moderate depression, and 10 (7.52%) had severe depression.

### Factors associated with antenatal depression:

Table 4 presents the results of a multivariate analysis examining the relationships between various participant characteristics and antenatal depression. The data included both unadjusted (crude) and adjusted odds ratios (ORs) along with 95% confidence intervals (CIs) for each characteristic.

**Table 4:** Multivariate analysis of factors associated with antenatal depression

Characteristic		No Antenatal Depression n (%)	Antenatal Depression n (%)	Unadjusted Analysis		Adjusted Analysis	
				cOR (95% CI)	*p*	aOR (95% CI)	*p*
Age	21 to 34	170(77.27)	50(22.73)	**Ref**		**Ref**	
	17 to 20	34(36.17)	60(63.83)	**6.00 (3.55–10.15)**	** < 0.001**	**7.74 (3.42–17.51)**	** < 0.001**
	35 and above	16(41.03)	23(58.97)	**4.89 (2.40–9.96)**	** < 0.001**	**3.16 (1.26–7.92)**	**0.014**
Marital status	Married/Cohabiting	214(65.24)	114(34.76)	**Ref**		Ref	
	Single/Separated	6(24.00)	19(76.0)	**5.94 (2.31–15.30)**	** < 0.001**	4.23 (0.65–27.66)	0.132
Education	Primary	118(54.13)	100(45.87)	Ref		Ref	
	Secondary/Tertiary	102(75.56)	33(24.44)	**0.38 (0.24–0.61)**	** < 0.001**	0.44 (0.20–1.00)	0.05
Employment	Employed	133(70.0)	57(30.0)	**Ref**		Ref	
	Unemployed	87(53.37)	76(46.63)	**2.04 (1.32–3.16)**	**0.001**	1.03 (0.50–2.13)	0.932
Household Income	≥ 500,000 UGX	110(69.62)	48(30.38)	**Ref**		Ref	
	< 500,000 UGX	110(56.41)	85(43.59)	**1.77 (1.14–2.75)**	**0.011**	1.38 (0.74–2.57)	0.306
Family mental illness history	No	193(63.70)	110(36.30)	Ref		Ref	
	Yes	27(54.0)	23(46)	1.49 (0.82–2.73)	0.192	1.71 (0.69–4.25)	0.245
History of violence	No	216(65.65)	113(34.35)	**Ref**		**Ref**	
	Yes	4(16.67)	20(83.33)	**9.56 (3.20–28.64)**	** < 0.001**	**6.51 (1.81–23.42)**	**0.004**
Alcohol use	No	215(66.77)	107(33.23)	**Ref**		**Ref**	
	Yes	5(16.13)	26(83.87)	**10.45 (3.90–27.97)**	** < 0.001**	**8.46 (2.76–25.95)**	** < 0.001**
Gestational Age	13 to 28 weeks	101(77.69)	29(22.31)	**Ref**		**Ref**	
	< 13 weeks	15(32.61)	31(67.39)	**7.20 (3.43–15.12)**	** < 0.001**	**4.49 (1.53–13.19)**	**0.006**
	> 28 weeks	104(58.76)	73(41.24)	**2.44 (1.47–4.07)**	**0.001**	1.90 (0.94–3.81)	0.075
Previous preeclampsia	No	209(63.33)	121(36.67)	Ref		**Ref**	
	Yes	11(47.83)	12(52.17)	1.88 (0.81–4.40)	0.143	**5.46 (1.69–17.63)**	**0.005**
History of abortion	No	205(68.56)	94(31.44)	**Ref**		**Ref**	
	Yes	15(27.78)	39(72.22)	**5.67 (2.98–10.79)**	** < 0.001**	**4.32 (1.88–9.95)**	**0.001**
Stillbirth	No	212(63.28)	123(36.72)	Ref		Ref	
	Yes	8(44.44)	10(55.56)	2.15 (0.83–5.60)	0.116	1.84 (0.46–7.31)	0.387
Pregnancy Planning	Yes (Planned)	138(87.90)	19(12.10)	**Ref**		**Ref**	
	No (Unplanned)	82(41.84)	114(58.16)	**10.10 (5.78–17.23)**	** < 0.001**	**12.59 (5.75–27.57)**	** < 0.001**

*Ref* Reference category, *cOR* crude odds ratio, *aOR* adjusted odds ratio, *CI* confidence interval, *p* p value, *UGX* Ugandan Shillings

## Discussion

### Objective 1: The prevalence of antenatal depression

It was determined to be 37.68%. This aligns with the worldwide prevalence range of 15 to 65%, as reported by Dadi and colleagues [4]. The joint prevalence of antenatal depression across 173 studies and 182 reports evaluated by systematic review and meta-analysis performed by Yin and colleagues was 20.7%, while the pooled prevalence of major antenatal depression across 72 studies and 79 reports was 15.0% [30]. The prevalence of antenatal depression in our study was higher than the worldwide average prevalence, and this disparity may be attributed to Uganda’s transition from a low- to a lower-middle-income country [31], and the prevalence of antenatal depression is significantly higher in low- or lower-middle-income countries [32].

Our findings also exceed the pooled prevalence of 27.01% for sub-Saharan Africa reported by Dadi and colleagues [1]. This variance might be due to cultural distinctions across various countries and the utilization of alternative assessment tools for antenatal depression other than the PHQ-9 in some studies.

The prevalence observed in this study is consistent with previous research conducted in Kenya, where the prevalence was 36% [33]. However, these findings are higher than those in Tanzania, where the prevalence was 11.5% [34], and in Rwanda, where it was 26.6% [35]. This discrepancy could be attributed to the fact that Tele and colleagues [33], as in our study, utilized the PHQ-9 as an assessment tool, whereas Ngocho and colleagues [34] and Umuziga and colleagues [35] used the Edinburgh Postnatal Depression Scale (EPDS). Different assessment tools may yield different prevalence rates due to variations in the way antenatal depression is measured.

The prevalence depicted aligns with results from northern Uganda, where it was reported as 35.8% [15]. However, this prevalence is notably higher than the 13% reported in a study conducted in Kamuli, Eastern Uganda [36]. This variation arises because the Kamuli study only considered moderate and severe depression [36].

The study findings also revealed that 70.68% of the participants with depression had mild symptoms, 21.80% had moderate symptoms, and 7.52% had severe symptoms. This emphasizes the importance of timely and targeted interventions to address the psychological needs of pregnant women and prevent the escalation of depression.

### Objective 2: Factors associated with antenatal depression among women attending antenatal care at MRRH

Younger women (≤ 20 years) and older women (≥ 35 years) were more likely to experience antenatal depression. These findings align with studies conducted in the Netherlands [37], Vietnam [38], and Uganda [39], suggesting that age plays a significant role in antenatal depression risk. Younger women may face challenges in coping with the demands of pregnancy and impending motherhood, while older women may have unique life circumstances and reproductive histories that contribute to depression [9, 33].

A history of domestic violence was strongly associated with antenatal depression, which is consistent with the findings of studies from South Asia [3], Bangalore [40], Zimbabwe [41], and Kenya [33]. These findings emphasize the detrimental effects of psychological and emotional abuse on maternal mental health during pregnancy. Addressing intimate partner violence and providing support to affected women are critical steps in preventing and managing antenatal depression.

Consistent with the findings of studies conducted in Ethiopia [27] and Kenya [33], alcohol use during pregnancy was significantly associated with antenatal depression. This highlights the potential exacerbation of maternal mental health concerns due to alcohol consumption. Health-care providers should prioritize alcohol screening and intervention during antenatal care to safeguard both maternal and fetal well-being.

Being in the first trimester of pregnancy was associated with antenatal depression. This aligns with the findings of studies from China and Kenya [42], indicating that early pregnancy may be characterized by heightened anxiety and mood fluctuations. Early identification and support for women in these situations are essential to prevent or manage antenatal depression.

A history of abortion was found to be significantly associated with antenatal depression in our study, which is consistent with findings from research conducted in Kenya [13]. This association suggested that previous experiences of abortion can have lasting emotional and psychological impacts on pregnant women.

Unplanned pregnancies were associated with higher odds of experiencing antenatal depression, which is consistent with the findings of other studies from the Netherlands [37], South Asia [3], Ethiopia [6], and Kenya [13]. These findings highlight the stress and uncertainty that unplanned pregnancies can cause. Integrating mental health screening and intervention programs tailored to women with unplanned pregnancies may alleviate the burden of antenatal depression.

In our study, a previous history of preeclampsia showed a moderate association with antenatal depression, although this association did not initially reach statistical significance in the unadjusted analysis. However, after implementing adjustments, the association became statistically significant, highlighting its relevance. These findings align with research from Canada [43], which suggested that a history of preeclampsia may increase the risk of experiencing depression.

## Conclusions

A significant prevalence of antenatal depression among pregnant women at MRRH was observed, emphasizing its importance as a public health concern.

Most cases of depression were classified as mild, followed by moderate and severe. This highlights the importance of appropriate and timely interventions to prevent further escalation of antenatal depression.

The identified associated factors included age, history of domestic violence, alcohol use, first-trimester pregnancy, abortion history, previous preeclampsia, and unplanned pregnancy.

### Study limitations

However, it is essential to acknowledge certain limitations of this study. The lack of data on physical activity and chronic infectious diseases may limit the generalizability of our results since both physical activity [44] and chronic infectious diseases have been linked to an increased prevalence of depression during pregnancy and the postpartum period [32].

The study's cross-sectional design limits its ability to establish causal relationships between the identified factors and antenatal depression. A longitudinal approach would have been useful for causal inference.

### Areas for further research

Longitudinal studies that follow pregnant women from early pregnancy to postpartum can provide valuable insights into the trajectory of antenatal depression and its long-term impacts on maternal and child health. Randomized controlled trials evaluating the effectiveness of different interventions for preventing and treating antenatal depression can provide evidence-based guidelines for clinical practice. Studies evaluating the integration of mental health services into routine antenatal care and assessing the feasibility and acceptability of such services are needed.

### Recommendations

We recommend integrating mental health screening and interventions into routine antenatal care to support pregnant women's well-being and maternal-child health. Antenatal care should include screening and intervention for domestic violence and substance abuse.

## Data Availability

The datasets used during the current study are available from the corresponding author upon request. Musa Kasujja via email: musakasujja2@gmail.com.

## Abbreviations

AAP: American Academy of Pediatrics
ACOG: American College of Obstetricians and Gynecologists
EPDS: Edinburgh Postnatal Depression Scale
MRRH: Mubende Regional Referral Hospital
PHQ 9: Patient Health Questionnaire- 9
